# Synthetic biology approaches to generate temperature‐sensitive alleles for the Sterile Insect Technique

**DOI:** 10.1111/1744-7917.70186

**Published:** 2025-11-03

**Authors:** Chun Yin Leung, Ernst A. Wimmer, Hassan M. M. Ahmed

**Affiliations:** ^1^ Department of Developmental Biology, Johann‐Friedrich‐Blumenbach‐Institute of Zoology and Anthropology, Göttingen Center of Molecular Biosciences University of Göttingen Göttingen Germany; ^2^ Department of Crop Protection Faculty of Agriculture – University of Khartoum Khartoum North Sudan

**Keywords:** CRISPR/Cas, genome editing, insect pest management, intein, N‐degron, protein design

## Abstract

The Sterile Insect Technique (SIT) is an environmentally friendly, sustainable pest control approach, which uses large‐scale releases of sterile insects to suppress or eradicate target populations through infertile matings. The efficiency of SIT is enhanced by male‐only releases requiring genetic sexing strains (GSSs) that are classically based on selectable recessive visible markers or temperature‐sensitive lethal (*tsl*) mutations and a rescue by a wild‐type allele translocated to the male‐determining chromosome. The transfer of identified or designed temperature‐sensitive alleles might allow the generation of neoclassical GSSs in additional SIT target species. By using precise genome‐editing tools, such as CRISPR/Cas, the creation of specific mutations in target genes and the integration of a wild‐type copy is feasible without the introduction of foreign DNA. This might ease regulation of neoclassical GSSs, since they are not considered transgenic. However, integration and expression of genes at male‐determining loci or chromosomes is not reliably established. Therefore, additional strategies to link temperature‐sensitive phenotypes to female development are required, which could be achieved by targeting genes involved in dosage compensation or sex determination. To create temperature‐sensitive alleles, rational protein design using advanced modeling and prediction tools to evaluate and tailor the effect of mutations on protein stability and temperature sensitivity can be used. In addition, emerging synthetic biology strategies such as temperature‐inducible N‐degrons or temperature‐sensitive inteins provide powerful tools to generate temperature sensitivity. Such approaches should enable conditional control over proteins causing female lethality or sex conversion and therefore promise straightforward generic approaches to generate GSSs for male‐only production in SIT target species.

## Graphical abstract

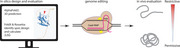

## Introduction

The Sterile Insect Technique (SIT) is an environmentally safe and effective pest management strategy, as it provides a species‐specific, sustainable pest control approach (Knipling, 1955) and is compatible with other pest control strategies of integrated pest management programs (Klassen & Vreysen, 2021). SIT was used successfully to eradicate insect pests such as the tsetse fly from Zanzibar, or the New World screwworm from Libya and the USA (Krafsur & Lindquist, 1996; Krafsur, 1998). SIT relies on the large‐scale release of sterile male insects that, through mating with wild females, suppress or even eradicate targeted populations (Knipling, 1955). Its successful integration into area‐wide insect pest control programs has now come of age (Dyck *et al.*, 2021). However, developing this technology for other insect pests remains a challenge. One particularly problematic area is the efficient separation of females from the sterile males before their release. Male‐only releases improve the economics of SIT and the efficiency of population suppression in the field (Rendón *et al.*, 2004; Franz *et al.*, 2021; Parker *et al.*, 2021). Removal of females would also limit the damage to fruits that could be caused by released females, or the potential risk of disease transmission in the case of mosquitoes, even if they are sterile (Wimmer, 2005; Alphey *et al.*, 2016). The best approach to remove females before release is through the use of so‐called genetic sexing strains (GSSs).

GSSs have been developed with classical genetics for multiple species, including the oriental fruit fly, *Bactrocera dorsalis* (McCombs & Saul, 1995), the Mediterranean fruit fly (medfly), *Ceratitis capitata* (Franz *et al.*, 2021), the Mexican fruit fly, *Anastrepha ludens* (Zepeda‐Cisneros *et al.*, 2014), the South American fruit fly, *Anastrepha fraterculus* (Meza *et al.*, 2020), and the mosquitoes, *Aedes aegypti* (Koskinioti *et al.*, 2020), *Anopheles albimanus* (Kaiser *et al.*, 1978), and *Anopheles arabiensis* (Yamada *et al.*, 2015). However, only the GSSs of *C. capitata* and *A. ludens* are used at large scale (Quintero‐Fong *et al.*, 2018; Franz *et al.*, 2021). They rely on a recessive visible marker or a conditional (temperature‐sensitive) lethal mutation. Females are homozygous for the mutated allele on autosomes, while males carry a wild‐type allele on the male‐determining chromosome, making them phenotypically wild‐type, given the recessive nature of the mutation (Franz *et al.*, 2021).

**Fig. 1:**
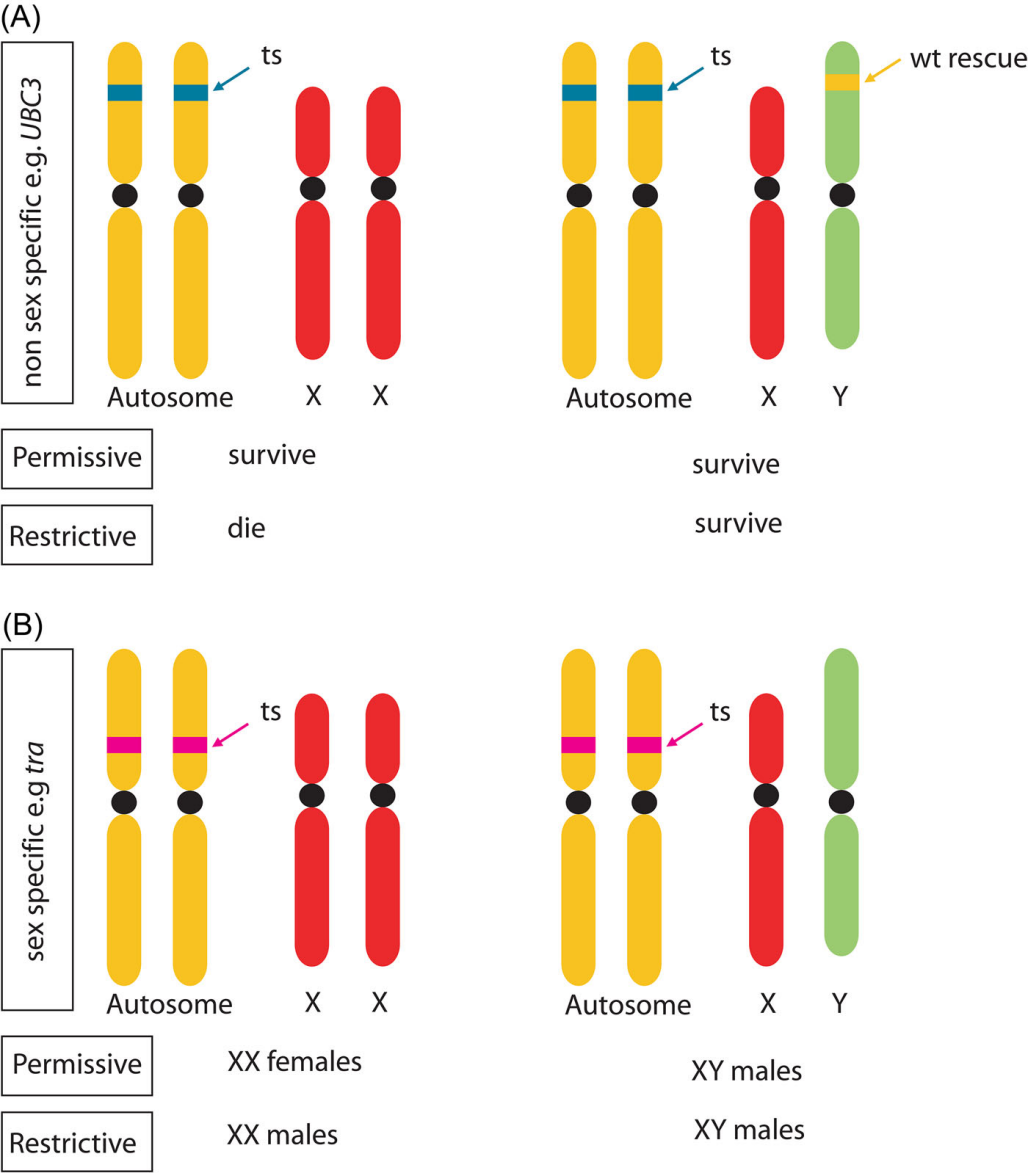
Genetic sexing systems using temperature‐sensitive (ts) alleles. (A) Genetic sexing strategy enables selective elimination of females based on a recessive ts lethal mutation at an autosomal locus (*e.g*., a temperature‐sensitive lethal [*tsl*] allele of *UBC3*). In homozygosity, the allele causes death at a restrictive temperature. A wild‐type rescue copy of the gene is inserted into the Y chromosome and therefore expressed only in males. At permissive temperatures, both sexes survive regardless of the genotype. At restrictive temperatures, females homozygous for the *tsl* allele die, while males carrying the Y‐linked rescue copy survive. Such a genetic sexing strain (GSS) can be reared in large quantities at the permissive temperature, switching to the restrictive temperature only in the final release generation. (B) Temperature‐dependent genetic sex conversion strategy. A ts allele is engineered for a key female sex‐determination gene, such as *transformer* (*tra*). At the permissive temperature, the ts allele is functional, allowing normal female development in XX individuals, resulting in a typical male‐to‐female sex ratio. At the restrictive temperature, the ts allele is not functional, disrupting female development and leading to the conversion of XX females into phenotypic XX males. This enables a conditional strategy for the production of male‐only release populations. No Y‐autosome translocation is required since the gene is only necessary for female development.

The classical genetic approach to develop GSSs usually consists of at least two principal components: an autosomal recessive selectable marker, which is necessary for sex separation or female lethality, and a translocation of the wild‐type allele to the male‐determining chromosome, which is required to link the inheritance of the functional allele of this marker to the male sex. GSSs based on pupal color mutation markers exist for several tephritid fruit fly species that include the *white pupae* (*wp*) marker for *C. capitata* (Franz *et al.*, 2021), *B. dorsalis* (McCombs & Saul, 1995), and *Zeugodacus cucurbitae* (McInnis *et al.*, 2004), as well as the *black pupae* marker for *A. ludens* (Zepeda‐Cisneros *et al.*, 2014), *A. fraterculus* (Meza *et al.*, 2020), *C. capitata* (Wappner *et al.*, 1996), and *Lucilia cuprina* (Whitten, 1969). A disadvantage of the pupal color markers is that females have to be reared along with the males up to the pupal stage, since sexing by sorting cannot be achieved before. Classical medfly GSSs deploy in addition to the *wp* marker a *temperature‐sensitive lethal* (*tsl*) mutation, which represents a selectable marker responsible for female killing. Again, a wild‐type allele of that genetic locus is translocated to the Y chromosome, rescuing males while females die from exposure to high temperature at embryonic stages (Fig. 1A). This represents a very cost‐effective way of producing male‐only medflies for SIT applications (Franz *et al.*, 2021). However, genes responsible for the *wp* or the *tsl* phenotypes have only recently been identified (Ward *et al.*, 2021; Sollazzo *et al.*, 2024; Aumann *et al.*, 2025), and it still needs to be determined how efficiently such mutations can be transferred to other species, as it has been shown so far only for the *wp* mutation from *C. capitata* to *Bactrocera tryoni* (Ward *et al.*, 2021).

Since the isolation of naturally occurring mutants is a labor‐intensive and serendipitous process, in which the time required to identify suitable genetic backgrounds is unpredictable, GSSs have only been developed for a few SIT target species so far. In this perspective article, we present synthetic biology approaches to design temperature‐sensitive alleles that could be introduced and evaluated in different insect pests of agricultural or public health importance.

### Temperature‐sensitive lethal alleles

 *tsl* alleles are conditional alleles of protein‐coding genes that cause lethality at the restrictive temperature while retaining normal function at the permissive temperature. The temperature dependency makes them invaluable tools in many fields including functional genetic analysis of essential genes (Horowitz, 1950; Fried, 1965) and applied biotechnology (Weber *et al.*, 2003). In the context of SIT and male‐only releases, *tsl* alleles offer a highly attractive solution to generate GSSs. However, regular laboratory screens for *tsl* phenotypes are usually carried out at two distinct temperatures within the regular temperature range of the species (Tasaka & Suzuki, 1973). Therefore, the restrictive temperature then lies within the span of temperatures the organism usually encounters. In the context of SIT, screens for *tsl* phenotypes are specifically conducted using a heat‐shock at a temperature outside the regular range (Ndo *et al.*, 2018). The *tsl* allele found in medfly has a restrictive temperature of 34 °C when embryos are exposed for at least 24 h (Franz *et al.*, 2021). This temperature, which medfly usually does not encounter for such a prolonged exposure time, thus lies outside its regular temperature range. Different approaches, such as screening in heterologous genetic systems like yeast, rational design based on protein structure predictions for destabilizing mutations, or other synthetic biology strategies using temperature‐sensitive protein modules like inteins or N‐degrons, should help to identify additional useful *tsl* alleles.

## Perspective

Since recent advances of precise genome‐editing tools, particularly the CRISPR/Cas system, have revolutionized the ability to design and introduce specific mutations into target genes also of nonmodel species (Inui *et al.*, 2014; Li & Handler, 2017; Aumann *et al.*, 2020), identified or designed *tsl* alleles could be introduced into the genome of insect agricultural pest or human disease vector species to generate GSSs for SIT strategies.

### Direct transfer of identified tsl mutations

Extensive efforts to identify the gene causing the *tsl* phenotype in the most successful GSS developed to date, the medfly strain VIENNA 8, have combined genomic, transcriptomic, bioinformatic, and cytogenetic approaches. In this way, 19 genes were identified that show sequence polymorphisms between a wild‐type strain and the GSS and have orthologs in *Drosophila melanogaster*, which are known to have temperature‐sensitive alleles (Sollazzo *et al.*, 2023).

### Deep orange

Among these identified genes was *vacuolar protein sorting‐associated protein 18 homolog* (*VPS18*), also known as *deep orange* (*dor*) in *D. melanogaster* (Sollazzo *et al.*, 2024). The gene is involved in autophagy and plays an important role in the docking, fusion, and trafficking of endosomes and lysosomes (Shestopal *et al.*, 1997; Lőrincz *et al.*, 2019). Since *dor* in medfly GSS has amino acid substitutions compared to wild‐type and the *dor* protein in *D. melanogaster* is known to harbor a number of temperature‐sensitive mutations that lead to lethality (Zhimulev *et al.*, 1981; Scott *et al.*, 1993; Shestopal *et al.*, 1997; Gailite *et al.*, 2012), *dor* was considered a key candidate gene causing the *tsl* phenotype in the VIENNA 8 GSS. In order to examine whether the candidate polymorphic mutation *dor^E839K^*, a lysine for a glutamate exchange at amino acid position 839, would be responsible for the *tsl* phenotype, the specific mutation was introduced into the medfly by CRISPR/Cas9‐mediated precise genome editing. Although this particular mutation did not cause the temperature‐sensitive lethality at 34 °C, indicating that this gene is probably not responsible for the *tsl* phenotype in the medfly GSSs, an unprecise event resulting in the E839K mutation along with a 51 duplication (*dor^51dup^*) causes *tsl* at 36 °C (Sollazzo *et al.*, 2024). Whether this particular mutation can be transferred to other tephritid fruit fly species needs to be tested, but based on the high conservation of the *dor* protein sequence, *dor* presents a promising candidate gene to generate *tsl* markers for GSSs in other insect species by precise genome editing.

### Lysyl‐tRNA synthetase

A continued and revised candidate screening to identify the gene locus in medfly GSSs finally revealed a mutation in the highly conserved *lysyl‐tRNA synthetase* (*LysRS*) gene as the cause for the *tsl* phenotype (Aumann *et al.*, 2025). *LysRS* encodes an essential enzyme that is part of the translational machinery in all living cells with homozygous mutations causing lethality (Freist & Gauss, 1995; Motzik *et al.*, 2013). *LysRS* in the GSSs carries a single nucleotide polymorphism that replaces histidine with tyrosine (H > Y substitution) in its core domain. By CRISPR/Cas9‐mediated precise genome editing, the H > Y substitution was introduced into a wild‐type non‐*tsl* medfly strain, and homozygous mutants showed the identical recessive phenotype as the GSSs with full embryonic lethality at 34 °C. Moreover, crossing the genome‐edited H > Y substitution mutation to GSSs resulted in fully susceptible temperature‐sensitive embryos, which revealed that the mutations do not complement each other and therefore must affect the same gene. In addition, a mini‐rescue gene (*mini‐LysRS*) was generated that can rescue the *tsl* phenotype when integrated into autosomes. With that, the necessary prerequisites are provided to start developing neoclassical GSSs for additional SIT target species by the precise genome editing of the highly conserved *LysRS* and the introduction of a potentially cisgenic rescue gene (Aumann *et al.*, 2025).

### Identification of tsl mutations in heterologous systems

For highly conserved genes, insect orthologs are often able to rescue the lack of a particular gene function in yeast. By such a complementation assay, alleles encoding different protein variants that show temperature sensitivity (nonfunctional at higher temperatures) can be isolated and characterized. For the identification or evaluation of *tsl* alleles of highly conserved genes at particular temperatures up to 37 °C, heterologous approaches, which involve the phenotypic rescue of mutants from the baker's yeast *Saccharomyces cerevisiae* through expression of an orthologous insect gene, can be used (Kuntamalla *et al.*, 2009).

### Protein kinase CK2

Casein kinase 2 (CK2) is a serine/threonine protein kinase that is highly conserved in eukaryotes, including yeast, mammals, and insects (Allende & Allende, 1995). CK2 consists of 2 catalytic α subunits and 2 regulatory *β* subunits and regulates cell proliferation, since the loss of CK2 function triggers cell cycle arrest at the transition stages between G1/S and G2/M phases (Hanna *et al.*, 1995). CK2 appears to be an essential protein in insects, since in *D. melanogaster*, a mutant allele of *dCK2α* called *Timekeeper* is recessive lethal (Lin *et al.*, 2002). By now, several temperature‐sensitive alleles of *dCK2α* have been isolated and characterized using a heterologous complementation system in *S. cerevisiae* (Kuntamalla *et al.*, 2009). The identified alleles successfully rescued the lethal phenotype of a CK2‐defective yeast strain at 29 °C, but failed to rescue at 35 or 37 °C. Of special interest is the amino acid substitution of an aspartate by an asparagine at position 212 (D212N), a highly conserved residue among serine/threonine protein kinases that is known to be involved in the stability of the activation subunit of CK2*α* (Kuntamalla *et al.*, 2009).

The mutation D212N causes temperature sensitivity with a restrictive temperature of 35 °C, which would be a suitable temperature for generating *tsl* GSSs in tephritid fruit flies. Since CK2*α* is highly conserved, respective mutations should be introduced into SIT target species by precise genome editing and evaluated for temperature sensitivity. In addition, other essential serine/threonine protein kinases can be assessed, whether temperature‐sensitive alleles could be generated by mutating the respective highly conserved aspartate.

### Ubiquitin‐conjugating enzymes

Target protein degradation is a crucial process in the regulation of various developmental programs, cell cycle progression, and maintenance of protein homeostasis. The ubiquitin‐conjugating enzymes (UBCs), also known as E2 enzymes, play a key role in the ubiquitin proteasome pathway. These enzymes work in concert with E1 (ubiquitin activating) and E3 (ubiquitin‐protein ligases) enzymes to attach chains of ubiquitin to the protein to be degraded by the proteasome proteolytic pathway of eukaryotic cells (Ciechanover, 1994). By selectively degrading regulatory proteins, UBCs help to control critical events, including mitosis, differentiation, and response to cellular stress. In *S. cerevisiae*, for *UBC3*, a temperature‐sensitive allele was obtained, resulting from a missense mutation that substitutes a highly conserved proline residue with serine (P > S) at the first position of a highly conserved “P‐X‐X‐P‐P” motif. This motif is present in nearly all UBCs and required for thermal stability as it stabilizes a specific turn in the enzyme (Ellison *et al.*, 1991). To generate temperature‐sensitive alleles also for other yeast UBCs, the conserved motif was systematically mutated to generate identical P > S amino acid substitution alleles in *UBC2* and *UBC9* (Ellison *et al.*, 1991; Betting & Seufert, 1996). The obtained *tsl* alleles showed restrictive temperatures between 34 and 39 °C.

UBCs are functionally conserved across eukaryotes, resulting in the ability of *D. melanogaster* UBC orthologs to rescue yeast mutants (Joanisse *et al.*, 1998). Since the “P‐X‐X‐P‐P” motif with the targeted proline is highly conserved in UBCs, analogous mutations should be transferable to SIT target species by precise genome editing and then evaluated for temperature sensitivity. In *D. melanogaster*, *UBC2* (Chen *et al.*, 2011), *UBC3* (Wu *et al.*, 2002), and *UBC9* (Epps & Tanda, 1998) represent recessive lethal genes, and respective mutations could thus cause temperature‐sensitive lethality. In the case that this also holds true for SIT target species, which needs to be assessed, targeting UBC orthologs could result in temperature‐sensitive alleles suitable for generating GSSs.

### Rescue of temperature‐sensitive lethality by wild‐type allele on male‐determining chromosome

To rescue the *tsl* phenotype caused by a recessive mutant allele of a specific gene, a wild‐type copy needs to be introduced to the male‐determining chromosome (Fig. 1A). However, classical approaches such as radiation need extensive screening to find suitable reciprocal translocations, which still result in semi‐sterility, since half of the gametes are genetically unbalanced (Franz *et al.*, 2021). More precise new generation genome‐editing tools have not yet reliably enabled the integration of genes into male‐determining loci or chromosomes, since the male‐determining locus usually resides in a predominantly heterochromatic region. Even in the model organism *D. melanogaster*, Y‐chromosomal integrations by genome editing have only been possible at the site of expressed genes and at a very low rate (Buchman & Akbari, 2019). Therefore, additional strategies need to be evaluated to link temperature‐sensitive phenotypes with female determination directly. One such strategy could be the generation of temperature‐sensitive alleles of genes involved in sex determination or dosage compensation (Fig. 1B).

### Targeting genes involved in insect sex determination or dosage compensation

Creating temperature‐sensitive alleles of genes involved in female sex determination can directly facilitate the generation of GSSs, eliminating the need to link a wild‐type allele to the male‐determining chromosome. In vinegar flies, the primary signal for female development is the female‐specific expression of *Sex‐lethal* (*Sxl*). In addition, as conserved in many insects, the genes *transformer* (*tra*) and *transformer*‐*2* (*tra2*) are also necessary for female development. Mutations in these genes resulting in nonfunctional proteins lead to female lethality, full sex conversion (turning females into functional XX males), or at the least turn females into harmless sterile intersex individuals (Cline, 1978, 1984; Skripsky & Lucchesi, 1982).

### Sex‐lethal

*Sxl* encodes an RNA‐binding protein and represents the primary signal for sex determination and the suppression of dosage compensation in females of *D. melanogaster* (Lucchesi & Skripsky, 1981; Cline, 1983, 1984; Gergen, 1987). *Sxl* is activated at early stages of embryogenesis only in embryos carrying a 1 : 1 ratio of X chromosomes to autosomes based on an X chromosome counting system (Erickson & Quintero, 2007). *Sxl* then functions primarily through the regulation of sex‐specific alternative splicing of specific RNA transcripts including its own pre‐mRNA and the downstream sex determination gene *tra* (reviewed in Penalva & Sánchez, 2003; Salz & Erickson, 2010; Salz, 2011; Venables *et al.*, 2012). In addition, *Sxl* controls the dosage compensation gene *male‐specific‐lethal 2* by translational repression (Gebauer *et al.*, 1998). In XX embryos, the Sxl promotes the female‐specific splicing of its own transcript by a positive feedback loop to ensure the continued production of functional Sxl. In contrast, in XY or XO embryos, male‐specific splicing of *Sxl* results in transcripts with a premature stop codon leading to a truncated nonfunctional Sxl (Cline, 1984; Bell *et al.*, 1991). Because *Sxl* is also required to suppress the XY(XO)‐specific dosage compensation in XX embryos by preventing hyperactivation of X chromosomes in females, nonfunctional alleles lead to female‐specific lethality when homozygous (Cline, 1978).

Due to its female‐specific necessity, *Sxl* represents an underexplored candidate to generate a GSS that does not require a rescue of its function in males. Therefore, creating an allele causing a *tsl* phenotype would be sufficient to generate a conditional GSS. Currently, there are no suitable *tsl* alleles available for *Sxl* in *D. melanogaster* or other species. Therefore, such *tsl* mutations will have to be generated by synthetic biology approaches as indicated below.

Targeting *Sxl* might be specific to vinegar flies only, for example, for the invasive fruit pest *D. suzukii* (Yan *et al.*, 2025), since *Sxl* does not have a conserved function in sex determination or dosage compensation across Diptera (Meise *et al.*, 1998). However, genes that are necessary for female‐specific repression of dosage compensation in other species—such as *female‐less* in *Anopheles gambiae* (Krzywinska *et al.*, 2021)—represent additional targets to potentially design female‐specific *tsl* alleles that could be assessed for the generation of conditional GSSs in further SIT target species.

### Transformer

 *tra* has been identified in several insect species and was found to play a central role in female sex determination (McKeown *et al.*, 1987; Pane *et al.*, 2002; Hediger *et al.*, 2010; Verhulst *et al.*, 2010). The gene is regulated at the mRNA splicing level, producing mRNA that translates into functional protein only in females. Male‐specific splicing leads to retention of male‐specific exons that introduce premature stop codons. In *D. melangaster*, *tra*‐splicing is controlled by *Sxl* (Boggs *et al.*, 1987), while in the housefly *Musca domestica* and in the medfly *C. capitata*, maternal contribution of tra protein (Tra) establishes an autoregulatory loop causing female continuity by sustaining female‐specific splicing (Pane *et al.*, 2002; Hediger *et al.*, 2010) that needs to be broken by a male‐determiner to enable male development (Sharma *et al.*, 2017; Meccariello *et al.*, 2019). In housefly, medfly, and other tephritid fruit flies, it has been shown that knockdown of *tra* by RNA interference results in sex conversion of XX females into fully functional fertile XX males (Pane *et al.*, 2002; Hediger *et al.*, 2010; Liu *et al.*, 2015; Laohakieat *et al.*, 2016). Therefore, *tra* represents a prime target gene to generate conditional GSSs based on sex conversion by causing a mutation in a single gene without the need of a rescue in males. However, similar to *Sxl*, no suitable *tsl* alleles are currently available, and respective mutations will have to be generated by synthetic biology approaches.

### Transformer‐2

*tra2* is a highly conserved gene that encodes a nuclear RNA‐binding protein (Tra2) involved in the regulation of pre‐mRNA splicing (Baker, 1989; Dauwalder *et al.*, 1996). In *D. melanogaster*, *tra2* plays a pivotal role in female somatic sex determination by formation of a Tra–Tra2 splicing complex that promotes female‐specific splicing of downstream genes such as *doublesex* (*dsx*) and *fruitless* (*fru*) (Baker, 1989; Heinrichs *et al.*, 1998). This leads to female‐specific protein isoforms, which in turn drive female development and behavior. In males, due to the absence of Tra protein, the Tra–Tra2 complex cannot be formed, and the genes *dsx* and *fru* undergo default male‐specific splicing, resulting in male development and behavior (McKeown *et al.*, 1987).

In other insects, such as *M. domestica* and *C. capitata*, the Tra–Tra2 complex is involved in the female‐specific splicing of *tra* pre‐mRNA as well as the downstream genes *dsx* and *fru*. In those species, it is thus involved in the establishment and maintenance of the *tra* autoregulatory loop. As for *tra*, also RNA interference of *tra2* results in sex conversion of XX females into fully functional fertile XX males in *M. domestica*, *C. capitata*, and other tephritid fruit flies (Burghardt *et al.*, 2005; Salvemini *et al.*, 2009; Liu *et al.*, 2015). Thus, also targeting *tra2* could lead to conditional GSSs based on a single gene mutation causing conditional sex conversion.

For *tra2*, 2 temperature‐sensitive missense mutations were isolated in *D. melanogaster* (Belote & Baker, 1982) that cause homozygous sterility at low temperatures (18−20 °C) and conversion of XX genotypes into intersex or pseudo‐male phenotypes at 29 °C (Amrein *et al.*, 1990). One of the temperature‐sensitive alleles was successfully introduced into the respective *tra2* orthologs of both *D. suzukii* and *C. capitata*. In *D. suzukii*, at the restrictive temperature of 26–29 °C, the XX individuals developed into sterile intersex and the XY as sterile males (Li & Handler, 2017). Since the nature of XY males’ sterility manifests in failure to produce sperm, the allele is not suitable for the generation of a GSS in this species. In the medfly *C. capitata*, the mutation resulted in fertile all‐male progeny at 19 °C, indicating that this temperature is still a restrictive temperature in this species (Aumann *et al.*, 2020). However, rearing medfly below 19 °C is not feasible. Therefore, the temperature‐sensitive alleles identified in *D. melanogaster* cannot be used in these pest species to generate GSSs.

### Synthetic biology strategies to generate temperature‐sensitive alleles

The failure in the transfer of *tra2* alleles to other species indicates the principle problem that temperature‐sensitive mutations derived from one species can have different permissive/restrictive temperatures and phenotypic manifestations in other species. This issue has also been observed for the gene *shibire* in in *B. tryoni* (Choo *et al.*, 2020) and seems a widespread phenomenon also described for nematode worms (Velayudhan & Ellis, 2022). Therefore, synthetic biology strategies such as rational design by identifying destabilizing mutations based on protein structure predictions or the use of temperature‐sensitive protein modules like inteins or N‐degrons should help to identify useful temperature‐sensitive alleles that can be introduced by precise genome editing, which then can be evaluated for temperature sensitivity in the SIT target species directly.

### Rational design

Unlike traditional random mutagenesis‐based approaches, rational design offers a precise alternative for the generation of temperature‐sensitive alleles (Varadarajan *et al.*, 1996; Chakshusmathi *et al.*, 2004; Poultney *et al.*, 2011). Rational design of temperature‐sensitive alleles uses structural and functional knowledge to introduce targeted point mutations that destabilize candidate proteins, which maintain activity at permissive temperatures but become nonfunctional at elevated temperatures. Common targets for rational design of temperature sensitivity are hydrophobic cores, catalytic sites, and protein–protein interaction surfaces leading to temperature‐dependent misfolding or degradation (Fletcher & Hamilton, 2006; Modarres *et al.*, 2016; Romero *et al.*, 2022). To be able to computationally design such alleles, it is imperative to have a reliable structure of the protein of interest (POI). Although not all structures of proteins have been determined by experimental structural biology means, in recent years, major progress in the field of protein structure prediction (Jumper *et al.*, 2021; Tunyasuvunakool *et al.*, 2021) and computational protein design (Butterfield *et al.*, 2017; Cao *et al.*, 2020) using artificial intelligence has been made. The development of reliable protein structure prediction tools such as AlphaFold2 and stability prediction platforms like Rosseta, Foldx, and I‐mutant enable precise computational prediction of the structure of the POI and the simulations of the effect of amino acid substitution on its folding and thermodynamic stability (Rohl *et al.*, 2004; Capriotti *et al.*, 2005; Schymkowitz *et al.*, 2005; Jumper *et al.*, 2021).

**Fig. 2:**
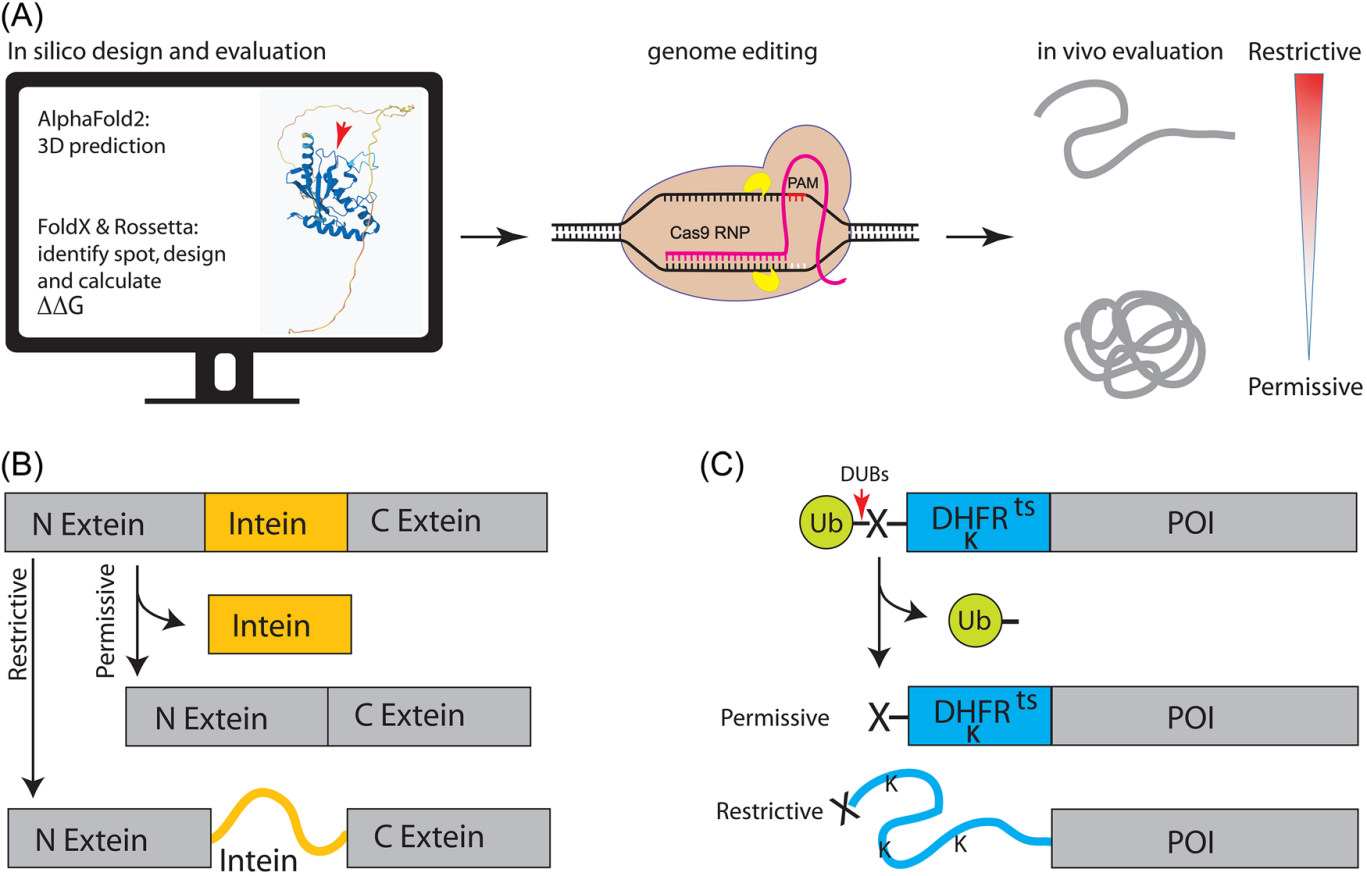
Strategies for the generation of temperature‐sensitive (ts) alleles. (A) Rational design. Computational workflow is used to predict structure‐based mutations in the protein of interest (POI). Protein structures are first predicted using AlphaFold2 (Jumper *et al.*, 2021), then subjected to stability analyses with FoldX and Rosetta (Rohl *et al.*, 2004; Schymkowitz *et al.*, 2005) to identify destabilizing mutations likely to confer temperature sensitivity. Destabilizing mutations are characterized by increase in the difference in Gibbs free energy (Δ*G*) between the wild‐type and mutant protein (ΔΔ*G* = Δ*G*_wt_ − Δ*G*_mut_). Selected point mutations are then introduced into the endogenous locus *via* precise genome editing. The resulting mutant strains are evaluated for phenotypic effects at permissive and restrictive temperatures, allowing identification of alleles with ts phenotypes. (B) A ts intein is inserted into an important domain of the POI to achieve conditional control of protein function through temperature‐controlled protein splicing. The target protein is expressed as an inactive precursor consisting of an N‐extein, a ts intein, and a C‐extein. At permissive temperatures, the intein folds correctly and catalyses its own excision *via* protein splicing, ligating the N‐ and C‐terminal exteins to produce a functional protein. At non‐permissive temperatures, the intein becomes misfolded and thus inactive, blocking splicing and preventing the formation of the active protein. (C) A temperature‐induced N‐degron is fused to the POI. This enables conditional degradation of the POI in response to elevated temperature *via* the N‐end rule pathway. The fusion protein consists of an N‐terminal ubiquitin moiety followed by a destabilizing residue (*e.g*., arginine), a ts dihydrofolate reductase (DHFR^ts^) domain, and the POI. During translation, the N‐terminal ubiquitin is cotranslationally cleaved by deubiquitinating enzymes (DUBs), exposing the engineered destabilizing residue (X; *e.g*., arginine), which is recognized by the N‐end rule pathway. At permissive temperatures, the DHFR^ts^ domain is folded, shielding internal lysine (K) residues critical for ubiquitination, and the fusion protein remains stable. Upon temperature shift to a restrictive condition, the DHFR^ts^ domain unfolds, exposing the hidden lysines (K), thereby enabling ubiquitination and proteasomal degradation.

A particularly effective strategy in the rational design of temperature‐sensitive alleles is targeting buried amino acids within the hydrophobic core of proteins (Varadarajan *et al.*, 1996; Chakshusmathi *et al.*, 2004). This strategy tremendously benefits from computational tools such as AlphaFold2, for structure prediction to identify buried residue (Jumper *et al.*, 2021) and the definition of secondary structure of proteins (DSSP) program to calculate solvent accessibility and estimate the energetic cost of mutations (Kabsch & Sander, 1983; Joosten *et al.*, 2010). However, it is also possible to predict burial status of residues based on the amino acid sequence of the protein (Varadarajan *et al.*, 1996; Chakshusmathi *et al.*, 2004). This sequence‐based prediction relies on the value of 2 parameters: the average hydrophobicity and the hydrophobic moment of each residue, with a stringent cutoff resulting in more than 80% accuracy. The predicted burial status can then be confirmed by the substitution of the respective amino acid residue with aspartate. If the burial status was predicted correctly, the substitution with aspartate should then lead to inactivation of the protein. Finally, 2–4 of the predicted buried residues will be substituted with lysine, serine, alanine, and tryptophan. All substitutions of the buried residues with different amino acids combinations will be tested for their temperature sensitivity. In Chakshusmathi *et al.* (2004), the authors were able to generate 17 tight temperature‐sensitive mutants of the *Escherichia coli* cytotoxin CcdB at 4 predicted buried residues. Additionally, they generated several temperature‐sensitive mutants of the yeast gene *Gal4* for which there was no report of temperature‐sensitive alleles before. Two of the *Gal4* mutants were used in *D. melanogaster* to establish temperature‐sensitive gene expression systems (Mondal *et al.*, 2007). Once promising candidate alleles are identified in respective target genes, for example, *Sxl*, *tra*, or *tra2*, the respective mutations can be introduced by genome‐editing tools in the SIT target species and the suitable permissive and restrictive temperatures validated (Fig. 2A).

### Temperature‐sensitive inteins

The word intein is short for “internal protein,” a self‐catalyzing intron‐like domain in a protein (Shah & Muir, 2014). Inteins perform a posttranslational autocatalytic protein‐splicing reaction removing themselves from the protein precursors and joining the N and C “external protein” (extein) parts (Kane *et al.*, 1990; Noren *et al.*, 2000) to generate intact functional proteins and therefore are considered introns of proteins. Temperature‐sensitive inteins lose their ability to excise themselves out of the host proteins under restrictive temperatures, leaving a precursor protein without function (Liang *et al.*, 2007). By introducing temperature‐sensitive inteins into critical regions of essential proteins, the autocatalytically splicing protein is conditionally produced in an active state at permissive temperatures, and in an inactive state at restrictive temperatures due to failure of the splice (Fig. 2B), as demonstrated for a repressor of Gal4 in *D. melanogaster* (Zeidler *et al.*, 2004). This mechanism offers a powerful tool for creating temperature‐controlled functionality of a certain protein. Whether the range of temperature sensitivity enables the generation of temperature‐sensitive alleles in target genes, such as *Sxl*, *tra*, or *tra2*, will have to be examined by introducing the respective inteins *via* genome‐editing tools in SIT target species and validate the suitable permissive and restrictive temperatures.

### Temperature‐inducible N‐degrons

An N‐degron is a type of degradation signal at the N‐terminus of proteins and dictates the protein half‐life through the N‐degron pathway, formerly called N‐end rule pathway (Varshavsky, 2024). According to this rule, the rate of protein degradation is linked to the identity of its N‐terminal amino acid residue. Several residues are considered as “destabilizing,” since they reduce the half‐life of proteins when they are located at their N‐terminus (Varshavsky, 1995). Besides the destabilizing residue, the degron consists of at least 1 internal lysine residue that serves as a site for polyubiquitination, thus flagging the protein for degradation by the ubiquitin proteasome pathway. Dohmen *et al.* (1994) have engineered an elegant, universal, and fusible temperature‐inducible degron that can be attached to theoretically any protein at its N‐terminus, rendering it temperature‐sensitive. This transferable degron consists of a thermolabile version of the mouse dihydrofolate reductase (DHFR^ts^) containing the amino acid substitution P67L, an N‐terminal destabilizing residue (arginine), and an ubiquitin moiety (Fig. 2C). Ubiquitin fusions are cleaved rapidly after the last amino acid of ubiquitin, enabling the generation of DHFR with a customized destabilizing N‐terminus residue such as arginine. Under permissive temperature, the DHFR^ts^ retains the normal half‐life even with the destabilizing residue at the N‐terminus, as its internal lysine residues, which serve as ubiquitination sites, are masked by its dense conformation. When it is exposed to a higher restrictive temperature, however, the conformation of the DHFR^ts^ is destabilized and the lysine residues are exposed, allowing rapid ubiquitination and subsequent proteasomal degradation of the DHFR^ts^ along with the targeted protein fusion (Dohmen *et al.*, 1994). This system enables long‐lived proteins at 23 °C that turn short‐lived at 37 °C. It has successfully been employed in the budding yeast *S. cerevisiae*, the fission yeast *Schizosaccharomyces pombe*, in the plants *Arabidopsis thaliana* and *Nicotiana benthamiana*, as well as in the genetic model insect *D. melanogaster*, with the restrictive temperature range being adjustable by introduction of 2 additional amino acid substitutions T39A and E173D (Faden *et al.*, 2016).

The transferable degron system can be fused to virtually any candidate gene for the purpose of creating temperature‐sensitive alleles of target genes such as *Sxl*, *tra*, or *tra2*. The suitable permissive and restrictive temperature ranges will then have to be experimentally assessed in target SIT species before respective GSSs can be established. Since the genes *DHFR* and *Ubiquitin* are highly conserved, it will be possible to engineer species‐specific temperature‐sensitive degrons using endogenous gene sequences and thus generate cisgenic conditional GSSs.

## Conclusion

Implementation of SIT, an integral component of area‐wide integrated pest management, can benefit tremendously from the ability to release only sterile males (Rendón *et al.*, 2004; Wimmer, 2005; Franz *et al.*, 2021; Parker *et al.*, 2021). This necessitates the elimination of the females before release. Ideally, females should be eliminated at an early embryonic stage or converted into males. Moreover, the female elimination or sex conversion needs to be conditional, as the respective GSS needs to be mass‐reared first before the sexing is applied in the release generation. Transgenic sexing strains (TSSs), in which conditionality is based on food supplements that suppress the sexing during rearing, have been generated for a variety of SIT target species, such as the medfly *C. capitata* (Ogaugwu *et al.*, 2013), the Caribbian and Mexican fruit flies *A. suspensa* and *A. ludens* (Schetelig & Handler, 2012; Schetelig *et al.*, 2016), the olive fruit fly *Bactrocera oleae* (Ant *et al.*, 2012), the New World screwworm *Cochliomyia hominivorax* (Concha *et al.*, 2016, 2020), the Australian sheep blow fly *L. cuprina* (Yan & Scott, 2015, 2020), the yellow fever mosquito *A. aegypti* (Fu *et al.*, 2010), as well as the pink bollworm *Pectinophora gossypiella*, and the diamondback moth *Plutella xylostella* (Jin *et al.*, 2013). Despite the generation of this broad spectrum of TSSs, none has so far been employed in area‐wide SIT release programs. This might have to do with the fact that they represent genetically modified organisms (GMOs), that underly strict regulations in many countries, and the public concerns related to the release of GMOs. Moreover, before TSSs could be used, they have to be evaluated under mass‐rearing conditions, which cannot be carried out at university or research institute settings. So far only 2 medfly TSS strains were evaluated under semi‐mass‐rearing conditions: VIENNA 8‐1260—a classical sexing strain bearing in addition transgenic fluorescent body and sperm markers (Scolari *et al.*, 2008)—showed lower productivity than the comparing VIENNA 8 strain (Rempoulakis *et al.*, 2016); and FSEL#32—based on conditional female‐specific embryonic lethality (Ogaugwu *et al.*, 2013)—showed a similar fecundity as a classical GSS, a higher production of male‐only pupae, but was inferior in respect to some other quality control parameters (Meza *et al.*, 2018). Moreover, several TSSs of *C. hominivorax*—also based on conditional female‐specific embryonic lethality—were tested under semi‐mass‐rearing conditions, which showed that at least some strains maintained sufficient fitness characteristics to be evaluated in mass‐rearing (Concha *et al.*, 2020). Furthermore, in mass‐rearing, there is a high potential for a genetic breakdown of the transgenic sexing system due to spontaneous mutations or the selection for suppressors (Handler, 2016; Zhao *et al.*, 2020). In addition, food‐supplemented conditionality—especially when based on antibiotics—has a potential negative impact on gut symbionts and therefore on the fitness of reared insects (Raymann *et al.*, 2017; Zhang *et al.*, 2022).

The use of temperature sensitivity as conditionality in GSSs has been proven to be very effective, but the lack of such *tsl* alleles in potential SIT target species has hampered the spread of this environmentally safe control strategy to fight additional insect pests. Classical methods of generating temperature‐sensitive alleles in model organisms are labor‐intensive, time‐consuming, and not cost‐effective, particularly for nonmodel organisms including pests of economic importance. New gene‐editing technologies based on CRISPR/Cas9 enable the precise recreation of temperature‐sensitive alleles. This “neoclassical” strategy (Petrucci *et al.*, 2025; Yan *et al.*, 2025) essentially replicates the “classical” genetic efforts and does not necessarily rely on the introduction of foreign DNA. Gene modification based on genome editing using site‐directed nucleases (SDNs) is categorized as SDN‐1, SDN‐2, or SDN‐3 depending on the means provided to repair the DNA double‐strand break: no repair template (SDN‐1), short nucleotide template to introduce few specific nucleotide changes (SDN‐2), and long DNA repair template to integrate additional DNA sequence at a selected genome location (Wray‐Cahen *et al.*, 2024). Neoclassical GSSs based on a single temperature‐sensitive allele can thus be classified as SDN‐2, which is already deregulated in several countries (Sprink & Wilhelm, 2024). GSSs relying on a *tsl* allele combined with a male‐determination‐linked wild‐type rescue allele could be generated in a cisgenic way falling under SDN‐3, which does not necessarily contain foreign DNA (Aumann *et al.*, 2025). Although GSSs generated by SDN‐3 are still regulated by the same framework as GMOs in the European Union (EFSA Panel on Genetically Modified Organisms (EFSA GMO Panel) et al., 2020), more and more countries around the world base classifications on the absence of foreign DNA in the final product (Sprink & Wilhelm, 2024), which could reduce regulation of neoclassical GSSs compared to GMOs.

Different approaches have been proposed and used to design temperature‐sensitive alleles which opens the door for their exploitation for the improvement of SIT and its expansion to new species. By using classical genetic methods to generate temperature‐sensitive alleles, the mutations are not known and their identification might take a long time (up to 30 years; Aumann *et al.*, 2025), which limits their transferability to other species. Using CRISPR/Cas‐based genome‐editing technology makes the recapitulation of promising phenotypes by engineering the orthologous genes in the target pest species straightforward. However, documented temperature‐sensitive mutations that can be recapitulated are limited and it is evident that the direct transfer of temperature‐sensitive mutations not always yield functional results (Li & Handler, 2017; Aumann *et al.*, 2020; Choo *et al.*, 2020). Additionally, a significant technical challenge is still the translocation of wild‐type rescue alleles to the male‐determining locus, which is often in a heterochromatic region.

Emerging synthetic biology strategies such as temperature‐inducible N‐degron pathways and temperature‐sensitive inteins are powerful tools to generate temperature‐sensitive alleles in sex‐determining or dosage compensation genes, which render the translocation of wild‐type rescue alleles to the male‐determining locus unnecessary. In addition to these synthetic biology strategies, recent advances in rational protein design together with sophisticated genome‐editing techniques have opened new avenues for precise engineering of temperature‐sensitive alleles. The method for rational protein design mentioned in Chakshusmathi *et al.* (2004) still requires intensive screening in living organisms to see if the designed proteins exhibit temperature sensitivity. However, the successful transfer of such temperature‐sensitive *Gal4* mutants from *S. cerevisiae* to *D. melanogaster* (Mondal *et al.*, 2007) indicates that this is in principle possible.

The use of advanced protein modeling tools such as Alphafold2, Foldx, and Rosseta (Rohl *et al.*, 2004; Schymkowitz *et al.*, 2005; Jumper *et al.*, 2021) to predict the structure of candidate gene products, to introduce informed mutations *in silico*, and to evaluate their effect on protein stability can significantly accelerate the design of temperature‐sensitive alleles. A promising strategy of rational design involves the introduction of specific mutations into genes based on their structural and functional characteristics, allowing for controlled inactivation of the gene product at restrictive temperatures. Integrating computational tools for the prediction of protein stability and folding can enhance the success rate of obtaining temperature‐sensitive alleles with suitable thermal sensitivity. By employing rational design principles, it is possible to tailor temperature‐sensitive alleles to target specific biological pathways such as sex determination, which is particularly useful in the context of SIT. These methods enable conditional control over protein levels involved in female lethality or sex conversion.

The growing body of knowledge in insect sex determination, combined with the rapid advancement in genome‐editing technologies, will enable the creation of new genetic sexing systems based on rational design of temperature‐sensitive alleles, or the use of temperature‐sensitive inteins or designed N‐degrons. These technologies promise more straightforward generic approaches to generate GSSs for male‐only production. As a result, they are expected to significantly enhance the scalability and efficiency of SIT and encourage its adoption for more insect species of agricultural, veterinary, and public health importance.

## Funding

Research funding was provided to HMMA by the Niedersächsisches Ministerium für Wissenschaft und Kultur within the program SPRUNG for research co‐operations between Lower Saxony and Israel. Publication costs for this study was provided by the International Atomic Energy Agency as part of the Coordinated Research Project “Generic approach for the development of genetic sexing strains for SIT applications.”

## Disclosure

The authors declare that they have no conflicts of interest associated with this work.
